# Supplementary material for: Pyruvate Dehydrogenase Kinase 1 inhibition mediated oxidative phosphorylation enhancement in cartilage promotes osteoarthritis progression
Source: BMC Musculoskelet Disord. 2023 Jul 20;24:597. doi: 10.1186/s12891-023-06585-6 (PMC10357736; doi:10.1186/s12891-023-06585-6)
Supplement: Supplementary file 1 — Additional file 1. [file 12891_2023_6585_MOESM1_ESM.zip › KEGG.Gsea.1653622432464/KEGG_AXON_GUIDANCE.html]

Details for gene set KEGG\_AXON\_GUIDANCE[GSEA]

|  || Dataset | 55457\_collapsed\_to\_symbols.cls.cls #OA\_versus\_normal.cls.cls #OA\_versus\_normal\_repos |
| Phenotype | cls.cls#OA\_versus\_normal\_repos |
| Upregulated in class | normal |
| GeneSet | KEGG\_AXON\_GUIDANCE |
| Enrichment Score (ES) | -0.29433864 |
| Normalized Enrichment Score (NES) | -1.2851425 |
| Nominal p-value | 0.08626198 |
| FDR q-value | 0.2983845 |
| FWER p-Value | 1.0 |
Table: GSEA Results Summary

  

Fig 1: Enrichment plot: KEGG\_AXON\_GUIDANCE      
 Profile of the Running ES Score & Positions of GeneSet Members on the Rank Ordered List

  

| SYMBOL | TITLE | RANK IN GENE LIST | RANK METRIC SCORE | RUNNING ES | CORE ENRICHMENT || 1 | NCK1 | NCK adaptor protein 1 [Source:HGNC Symbol;Acc:HGNC:7664] | 222 | 0.804 | 0.0075 | No |
| 2 | UNC5C | unc-5 netrin receptor C [Source:HGNC Symbol;Acc:HGNC:12569] | 486 | 0.664 | 0.0074 | No |
| 3 | EPHA3 | EPH receptor A3 [Source:HGNC Symbol;Acc:HGNC:3387] | 553 | 0.641 | 0.0221 | No |
| 4 | MAPK3 | mitogen-activated protein kinase 3 [Source:HGNC Symbol;Acc:HGNC:6877] | 757 | 0.579 | 0.0241 | No |
| 5 | EFNA4 | ephrin A4 [Source:HGNC Symbol;Acc:HGNC:3224] | 871 | 0.550 | 0.0323 | No |
| 6 | SEMA3D | semaphorin 3D [Source:HGNC Symbol;Acc:HGNC:10726] | 1257 | 0.466 | 0.0166 | No |
| 7 | SEMA4F | ssemaphorin 4F [Source:HGNC Symbol;Acc:HGNC:10734] | 1639 | 0.407 | -0.0006 | No |
| 8 | EPHA7 | EPH receptor A7 [Source:HGNC Symbol;Acc:HGNC:3390] | 1770 | 0.388 | 0.0012 | No |
| 9 | SEMA3B | semaphorin 3B [Source:HGNC Symbol;Acc:HGNC:10724] | 1777 | 0.388 | 0.0127 | No |
| 10 | DCC | DCC netrin 1 receptor [Source:HGNC Symbol;Acc:HGNC:2701] | 1862 | 0.378 | 0.0178 | No |
| 11 | RASA1 | RAS p21 protein activator 1 [Source:HGNC Symbol;Acc:HGNC:9871] | 2506 | 0.302 | -0.0231 | No |
| 12 | SEMA3C | semaphorin 3C [Source:HGNC Symbol;Acc:HGNC:10725] | 2741 | 0.276 | -0.0329 | No |
| 13 | NFATC1 | nuclear factor of activated T cells 1 [Source:HGNC Symbol;Acc:HGNC:7775] | 3027 | 0.247 | -0.0476 | No |
| 14 | ROBO3 | roundabout guidance receptor 3 [Source:HGNC Symbol;Acc:HGNC:13433] | 3069 | 0.243 | -0.0432 | No |
| 15 | NFATC3 | nuclear factor of activated T cells 3 [Source:HGNC Symbol;Acc:HGNC:7777] | 3079 | 0.242 | -0.0365 | No |
| 16 | SEMA3A | semaphorin 3A [Source:HGNC Symbol;Acc:HGNC:10723] | 3104 | 0.239 | -0.0310 | No |
| 17 | RGS3 | regulator of G protein signaling 3 [Source:HGNC Symbol;Acc:HGNC:9999] | 3247 | 0.226 | -0.0351 | No |
| 18 | SEMA5A | semaphorin 5A [Source:HGNC Symbol;Acc:HGNC:10736] | 3308 | 0.220 | -0.0330 | No |
| 19 | CDK5 | cyclin dependent kinase 5 [Source:HGNC Symbol;Acc:HGNC:1774] | 3400 | 0.211 | -0.0335 | No |
| 20 | EPHB2 | EPH receptor B2 [Source:HGNC Symbol;Acc:HGNC:3393] | 3677 | 0.188 | -0.0493 | No |
| 21 | GNAI3 | G protein subunit alpha i3 [Source:HGNC Symbol;Acc:HGNC:4387] | 3694 | 0.186 | -0.0448 | No |
| 22 | NRP1 | neuropilin 1 [Source:HGNC Symbol;Acc:HGNC:8004] | 3704 | 0.185 | -0.0398 | No |
| 23 | ROBO1 | roundabout guidance receptor 1 [Source:HGNC Symbol;Acc:HGNC:10249] | 3737 | 0.182 | -0.0367 | No |
| 24 | PPP3CA | protein phosphatase 3 catalytic subunit alpha [Source:HGNC Symbol;Acc:HGNC:9314] | 3803 | 0.176 | -0.0363 | No |
| 25 | NTN3 | netrin 3 [Source:HGNC Symbol;Acc:HGNC:8030] | 3840 | 0.172 | -0.0338 | No |
| 26 | RAC3 | Rac family small GTPase 3 [Source:HGNC Symbol;Acc:HGNC:9803] | 3849 | 0.171 | -0.0292 | No |
| 27 | PAK1 | p21 (RAC1) activated kinase 1 [Source:HGNC Symbol;Acc:HGNC:8590] | 3861 | 0.169 | -0.0248 | No |
| 28 | PLXNC1 | plexin C1 [Source:HGNC Symbol;Acc:HGNC:9106] | 4112 | 0.145 | -0.0399 | No |
| 29 | PLXNB2 | plexin B2 [Source:HGNC Symbol;Acc:HGNC:9104] | 4301 | 0.128 | -0.0506 | No |
| 30 | UNC5B | unc-5 netrin receptor B [Source:HGNC Symbol;Acc:HGNC:12568] | 4352 | 0.124 | -0.0507 | No |
| 31 | PLXNB3 | plexin B3 [Source:HGNC Symbol;Acc:HGNC:9105] | 4493 | 0.113 | -0.0582 | No |
| 32 | EPHB6 | EPH receptor B6 [Source:HGNC Symbol;Acc:HGNC:3396] | 4721 | 0.094 | -0.0730 | No |
| 33 | SEMA3E | semaphorin 3E [Source:HGNC Symbol;Acc:HGNC:10727] | 4815 | 0.086 | -0.0776 | No |
| 34 | SLIT3 | slit guidance ligand 3 [Source:HGNC Symbol;Acc:HGNC:11087] | 5134 | 0.061 | -0.1006 | No |
| 35 | SEMA3G | semaphorin 3G [Source:HGNC Symbol;Acc:HGNC:30400] | 5260 | 0.051 | -0.1088 | No |
| 36 | PPP3CB | protein phosphatase 3 catalytic subunit beta [Source:HGNC Symbol;Acc:HGNC:9315] | 5311 | 0.046 | -0.1113 | No |
| 37 | L1CAM | L1 cell adhesion molecule [Source:HGNC Symbol;Acc:HGNC:6470] | 5512 | 0.032 | -0.1259 | No |
| 38 | EPHB3 | EPH receptor B3 [Source:HGNC Symbol;Acc:HGNC:3394] | 5614 | 0.023 | -0.1331 | No |
| 39 | NTNG1 | netrin G1 [Source:HGNC Symbol;Acc:HGNC:23319] | 5627 | 0.022 | -0.1334 | No |
| 40 | DPYSL2 | dihydropyrimidinase like 2 [Source:HGNC Symbol;Acc:HGNC:3014] | 5839 | 0.006 | -0.1497 | No |
| 41 | RAC2 | Rac family small GTPase 2 [Source:HGNC Symbol;Acc:HGNC:9802] | 6098 | -0.013 | -0.1695 | No |
| 42 | EFNA2 | ephrin A2 [Source:HGNC Symbol;Acc:HGNC:3222] | 6353 | -0.032 | -0.1884 | No |
| 43 | CXCR4 | C-X-C motif chemokine receptor 4 [Source:HGNC Symbol;Acc:HGNC:2561] | 6382 | -0.034 | -0.1895 | No |
| 44 | FES | "FES proto-oncogene, tyrosine kinase [Source:HGNC Symbol;Acc:HGNC:3657]" | 6491 | -0.042 | -0.1967 | No |
| 45 | SEMA4A | semaphorin 4A [Source:HGNC Symbol;Acc:HGNC:10729] | 6586 | -0.049 | -0.2025 | No |
| 46 | ARHGEF12 | Rho guanine nucleotide exchange factor 12 [Source:HGNC Symbol;Acc:HGNC:14193] | 6587 | -0.049 | -0.2010 | No |
| 47 | MAPK1 | mitogen-activated protein kinase 1 [Source:HGNC Symbol;Acc:HGNC:6871] | 6853 | -0.068 | -0.2197 | No |
| 48 | EFNA5 | ephrin A5 [Source:HGNC Symbol;Acc:HGNC:3225] | 6997 | -0.080 | -0.2284 | No |
| 49 | PPP3CC | protein phosphatase 3 catalytic subunit gamma [Source:HGNC Symbol;Acc:HGNC:9316] | 7031 | -0.082 | -0.2284 | No |
| 50 | EFNB1 | ephrin B1 [Source:HGNC Symbol;Acc:HGNC:3226] | 7079 | -0.086 | -0.2294 | No |
| 51 | SRGAP3 | SLIT-ROBO Rho GTPase activating protein 3 [Source:HGNC Symbol;Acc:HGNC:19744] | 7091 | -0.087 | -0.2276 | No |
| 52 | RAC1 | Rac family small GTPase 1 [Source:HGNC Symbol;Acc:HGNC:9801] | 7192 | -0.096 | -0.2325 | No |
| 53 | RHOD | ras homolog family member D [Source:HGNC Symbol;Acc:HGNC:670] | 7202 | -0.096 | -0.2302 | No |
| 54 | NRAS | "NRAS proto-oncogene, GTPase [Source:HGNC Symbol;Acc:HGNC:7989]" | 7300 | -0.102 | -0.2346 | No |
| 55 | PTK2 | protein tyrosine kinase 2 [Source:HGNC Symbol;Acc:HGNC:9611] | 7315 | -0.103 | -0.2325 | No |
| 56 | MET | "MET proto-oncogene, receptor tyrosine kinase [Source:HGNC Symbol;Acc:HGNC:7029]" | 7322 | -0.104 | -0.2298 | No |
| 57 | PAK5 | p21 (RAC1) activated kinase 5 [Source:HGNC Symbol;Acc:HGNC:15916] | 7558 | -0.124 | -0.2444 | No |
| 58 | PAK2 | p21 (RAC1) activated kinase 2 [Source:HGNC Symbol;Acc:HGNC:8591] | 7762 | -0.139 | -0.2560 | No |
| 59 | EPHA1 | EPH receptor A1 [Source:HGNC Symbol;Acc:HGNC:3385] | 7771 | -0.139 | -0.2523 | No |
| 60 | SEMA4D | semaphorin 4D [Source:HGNC Symbol;Acc:HGNC:10732] | 7780 | -0.140 | -0.2486 | No |
| 61 | PLXNA3 | plexin A3 [Source:HGNC Symbol;Acc:HGNC:9101] | 7794 | -0.141 | -0.2452 | No |
| 62 | HRAS | "HRas proto-oncogene, GTPase [Source:HGNC Symbol;Acc:HGNC:5173]" | 7977 | -0.153 | -0.2547 | No |
| 63 | GNAI1 | G protein subunit alpha i1 [Source:HGNC Symbol;Acc:HGNC:4384] | 8110 | -0.164 | -0.2600 | No |
| 64 | SRGAP2 | SLIT-ROBO Rho GTPase activating protein 2 [Source:HGNC Symbol;Acc:HGNC:19751] | 8116 | -0.164 | -0.2553 | No |
| 65 | EFNA3 | ephrin A3 [Source:HGNC Symbol;Acc:HGNC:3223] | 8194 | -0.171 | -0.2561 | No |
| 66 | PAK3 | p21 (RAC1) activated kinase 3 [Source:HGNC Symbol;Acc:HGNC:8592] | 8266 | -0.176 | -0.2562 | No |
| 67 | EPHB1 | EPH receptor B1 [Source:HGNC Symbol;Acc:HGNC:3392] | 8342 | -0.181 | -0.2564 | No |
| 68 | SLIT2 | slit guidance ligand 2 [Source:HGNC Symbol;Acc:HGNC:11086] | 8449 | -0.190 | -0.2588 | No |
| 69 | CHP2 | calcineurin like EF-hand protein 2 [Source:HGNC Symbol;Acc:HGNC:24927] | 8573 | -0.199 | -0.2623 | No |
| 70 | RHOA | ras homolog family member A [Source:HGNC Symbol;Acc:HGNC:667] | 8601 | -0.201 | -0.2582 | No |
| 71 | GNAI2 | G protein subunit alpha i2 [Source:HGNC Symbol;Acc:HGNC:4385] | 8686 | -0.208 | -0.2583 | No |
| 72 | PAK6 | p21 (RAC1) activated kinase 6 [Source:HGNC Symbol;Acc:HGNC:16061] | 9068 | -0.237 | -0.2808 | No |
| 73 | ABL1 | "ABL proto-oncogene 1, non-receptor tyrosine kinase [Source:HGNC Symbol;Acc:HGNC:76]" | 9081 | -0.238 | -0.2744 | No |
| 74 | NTN1 | netrin 1 [Source:HGNC Symbol;Acc:HGNC:8029] | 9276 | -0.254 | -0.2817 | No |
| 75 | SEMA7A | semaphorin 7A (John Milton Hagen blood group) [Source:HGNC Symbol;Acc:HGNC:10741] | 9314 | -0.256 | -0.2767 | No |
| 76 | EPHA5 | EPH receptor A5 [Source:HGNC Symbol;Acc:HGNC:3389] | 9541 | -0.277 | -0.2858 | Yes |
| 77 | ABLIM1 | actin binding LIM protein 1 [Source:HGNC Symbol;Acc:HGNC:78] | 9607 | -0.284 | -0.2821 | Yes |
| 78 | EFNA1 | ephrin A1 [Source:HGNC Symbol;Acc:HGNC:3221] | 9687 | -0.290 | -0.2793 | Yes |
| 79 | PLXNA1 | plexin A1 [Source:HGNC Symbol;Acc:HGNC:9099] | 9730 | -0.294 | -0.2735 | Yes |
| 80 | LIMK1 | LIM domain kinase 1 [Source:HGNC Symbol;Acc:HGNC:6613] | 9746 | -0.296 | -0.2655 | Yes |
| 81 | NCK2 | NCK adaptor protein 2 [Source:HGNC Symbol;Acc:HGNC:7665] | 9755 | -0.297 | -0.2569 | Yes |
| 82 | LIMK2 | LIM domain kinase 2 [Source:HGNC Symbol;Acc:HGNC:6614] | 9812 | -0.302 | -0.2520 | Yes |
| 83 | CXCL12 | C-X-C motif chemokine ligand 12 [Source:HGNC Symbol;Acc:HGNC:10672] | 9901 | -0.312 | -0.2492 | Yes |
| 84 | PAK4 | p21 (RAC1) activated kinase 4 [Source:HGNC Symbol;Acc:HGNC:16059] | 10004 | -0.322 | -0.2473 | Yes |
| 85 | FYN | "FYN proto-oncogene, Src family tyrosine kinase [Source:HGNC Symbol;Acc:HGNC:4037]" | 10092 | -0.330 | -0.2439 | Yes |
| 86 | EPHB4 | EPH receptor B4 [Source:HGNC Symbol;Acc:HGNC:3395] | 10097 | -0.330 | -0.2340 | Yes |
| 87 | EPHA2 | EPH receptor A2 [Source:HGNC Symbol;Acc:HGNC:3386] | 10219 | -0.343 | -0.2328 | Yes |
| 88 | EFNB3 | ephrin B3 [Source:HGNC Symbol;Acc:HGNC:3228] | 10232 | -0.345 | -0.2231 | Yes |
| 89 | EFNB2 | ephrin B2 [Source:HGNC Symbol;Acc:HGNC:3227] | 10298 | -0.350 | -0.2174 | Yes |
| 90 | SEMA6C | semaphorin 6C [Source:HGNC Symbol;Acc:HGNC:10740] | 10508 | -0.374 | -0.2222 | Yes |
| 91 | KRAS | "KRAS proto-oncogene, GTPase [Source:HGNC Symbol;Acc:HGNC:6407]" | 10544 | -0.377 | -0.2132 | Yes |
| 92 | ROCK2 | Rho associated coiled-coil containing protein kinase 2 [Source:HGNC Symbol;Acc:HGNC:10252] | 10648 | -0.388 | -0.2093 | Yes |
| 93 | PLXNB1 | plexin B1 [Source:HGNC Symbol;Acc:HGNC:9103] | 10682 | -0.391 | -0.1998 | Yes |
| 94 | CHP1 | calcineurin like EF-hand protein 1 [Source:HGNC Symbol;Acc:HGNC:17433] | 10940 | -0.423 | -0.2068 | Yes |
| 95 | SEMA6A | semaphorin 6A [Source:HGNC Symbol;Acc:HGNC:10738] | 11126 | -0.446 | -0.2075 | Yes |
| 96 | GSK3B | glycogen synthase kinase 3 beta [Source:HGNC Symbol;Acc:HGNC:4617] | 11157 | -0.451 | -0.1959 | Yes |
| 97 | SEMA6B | semaphorin 6B [Source:HGNC Symbol;Acc:HGNC:10739] | 11186 | -0.456 | -0.1840 | Yes |
| 98 | PLXNA2 | plexin A2 [Source:HGNC Symbol;Acc:HGNC:9100] | 11254 | -0.465 | -0.1749 | Yes |
| 99 | NFATC4 | nuclear factor of activated T cells 4 [Source:HGNC Symbol;Acc:HGNC:7778] | 11363 | -0.481 | -0.1685 | Yes |
| 100 | ABLIM3 | actin binding LIM protein family member 3 [Source:HGNC Symbol;Acc:HGNC:29132] | 11398 | -0.487 | -0.1561 | Yes |
| 101 | SEMA4C | semaphorin 4C [Source:HGNC Symbol;Acc:HGNC:10731] | 11533 | -0.505 | -0.1509 | Yes |
| 102 | SEMA6D | semaphorin 6D [Source:HGNC Symbol;Acc:HGNC:16770] | 11672 | -0.528 | -0.1454 | Yes |
| 103 | SEMA3F | semaphorin 3F [Source:HGNC Symbol;Acc:HGNC:10728] | 11706 | -0.534 | -0.1315 | Yes |
| 104 | EPHA4 | EPH receptor A4 [Source:HGNC Symbol;Acc:HGNC:3388] | 11863 | -0.563 | -0.1263 | Yes |
| 105 | ROCK1 | Rho associated coiled-coil containing protein kinase 1 [Source:HGNC Symbol;Acc:HGNC:10251] | 12046 | -0.605 | -0.1218 | Yes |
| 106 | SLIT1 | slit guidance ligand 1 [Source:HGNC Symbol;Acc:HGNC:11085] | 12066 | -0.611 | -0.1044 | Yes |
| 107 | SEMA4G | semaphorin 4G [Source:HGNC Symbol;Acc:HGNC:10735] | 12131 | -0.626 | -0.0901 | Yes |
| 108 | CFL1 | cofilin 1 [Source:HGNC Symbol;Acc:HGNC:1874] | 12352 | -0.691 | -0.0859 | Yes |
| 109 | ITGB1 | integrin subunit beta 1 [Source:HGNC Symbol;Acc:HGNC:6153] | 12566 | -0.780 | -0.0785 | Yes |
| 110 | RND1 | Rho family GTPase 1 [Source:HGNC Symbol;Acc:HGNC:18314] | 12589 | -0.790 | -0.0558 | Yes |
| 111 | PPP3R1 | "protein phosphatase 3 regulatory subunit B, alpha [Source:HGNC Symbol;Acc:HGNC:9317]" | 12653 | -0.829 | -0.0351 | Yes |
| 112 | CDC42 | cell division cycle 42 [Source:HGNC Symbol;Acc:HGNC:1736] | 12681 | -0.852 | -0.0109 | Yes |
| 113 | NFAT5 | nuclear factor of activated T cells 5 [Source:HGNC Symbol;Acc:HGNC:7774] | 12741 | -0.903 | 0.0124 | Yes |
Table: GSEA details [plain text format]

  

Fig 2: KEGG\_AXON\_GUIDANCE      
 Blue-Pink O' Gram in the Space of the Analyzed GeneSet

  

Fig 3: KEGG\_AXON\_GUIDANCE: Random ES distribution      
 Gene set null distribution of ES for **KEGG\_AXON\_GUIDANCE**
